# Identification of a novel variant of the ciliopathic gene *FUZZY* associated with craniosynostosis

**DOI:** 10.1038/s41431-021-00988-6

**Published:** 2021-11-01

**Authors:** William B. Barrell, Hadeel Adel Al-Lami, Jacqueline A. C. Goos, Sigrid M. A. Swagemakers, Marieke van Dooren, Elena Torban, Peter J. van der Spek, Irene M. J. Mathijssen, Karen J. Liu

**Affiliations:** 1 Centre for Craniofacial and Regenerative Biology, King’s College London, London, SE1 9RT UK; 2 Department of Orthodontics, College of Dentistry, University of Baghdad, Baghdad, Iraq; 3 Department of Plastic and Reconstructive Surgery and Hand Surgery, Erasmus University Medical Centre, Rotterdam, The Netherlands; 4 Department of Bioinformatics, Erasmus University Medical Centre, Rotterdam, The Netherlands; 5 Department of Clinical Genetics, Erasmus University Medical Centre, Rotterdam, The Netherlands; 6 Department of Medicine, McGill University Health Centre, Montreal, Canada

**Keywords:** Development, Disease model, Mutation

## Abstract

Craniosynostosis is a birth defect occurring in approximately one in 2000 live births, where premature fusion of the cranial bones inhibits growth of the skull during critical periods of brain development. The resulting changes in skull shape can lead to compression of the brain, causing severe complications. While we have some understanding of the molecular pathology of craniosynostosis, a large proportion of cases are of unknown genetic aetiology. Based on studies in mouse, we previously proposed that the ciliopathy gene *Fuz* should be considered a candidate craniosynostosis gene. Here, we report a novel variant of *FUZ* (*c.851* G > C, p.(Arg284Pro)) found in monozygotic twins presenting with craniosynostosis. To investigate whether *Fuz* has a direct role in regulating osteogenic fate and mineralisation, we cultured primary osteoblasts and mouse embryonic fibroblasts (MEFs) from *Fuz* mutant mice. Loss of *Fuz* resulted in increased osteoblastic mineralisation. This suggests that FUZ protein normally acts as a negative regulator of osteogenesis. We then used *Fuz* mutant MEFs, which lose functional primary cilia, to test whether the FUZ p.(Arg284Pro) variant could restore FUZ function during ciliogenesis. We found that expression of the FUZ p.(Arg284Pro) variant was sufficient to partially restore cilia numbers, but did not mediate a comparable response to Hedgehog pathway activation. Together, this suggests the osteogenic effects of FUZ p.(Arg284Pro) do not depend upon initiation of ciliogenesis.

## Introduction

Craniosynostosis is the premature fusion of one or more sutures of the skull vault and has an incidence of (approximately) 1:2000 live births [1–3]. While there are syndromes that present with craniosynostosis (sometimes with known genetic aetiology), the genetic associations of the more common non-syndromic craniosynostosis (approximately 85%) is less well understood [4]. Craniosynostosis is closely linked to the development of the cranial bones and sutures. During embryogenesis, there are condensations of neural crest (broadly anterior) and mesodermally (broadly posterior) derived skeletal progenitors. These cells then proliferate and differentiate into osteoblasts that go on to lay down matrix (osteoid). This matrix is then mineralised to form bone. Between the growth front of these bones the mesenchymal sutures work to prevent premature fusion. Pathological fusion can result from aberrant specification of the sutural mesenchyme towards the bone lineage, increased osteoblastic/decreased osteoclastic activity, or from aberrant mechanical forces causing bone fusion at close approximating surfaces [5]. Craniosynostosis can lead to raised intracranial pressure, which can impair eyesight and mental development [1, 6, 7]. There are good surgical treatment options for craniosynostosis; however, genetic sequencing is crucial for our understanding of the developmental aetiology of this disorder.

**Fig. 1:**
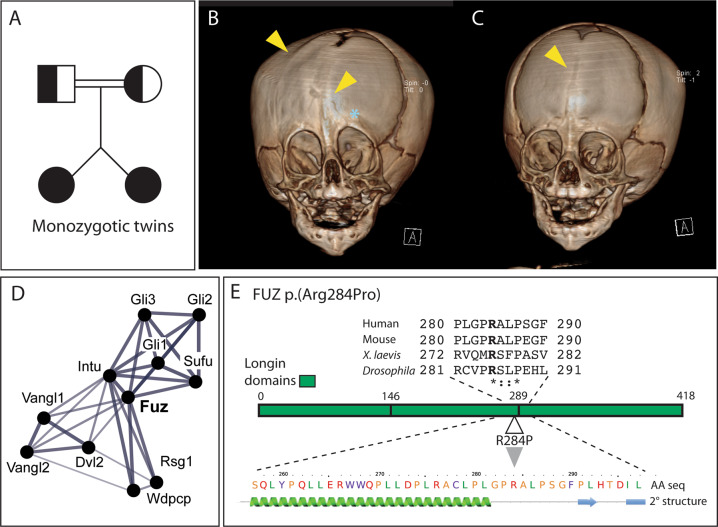
Novel missense variant: FUZ p.(Arg284Pro). Family pedigree scheme (**A**). Computed tomography (CT) scan reconstructions of twins exhibiting metopic craniosynostosis (**B** and **C**, yellow arrowhead, midline). The twin in (**B**) also exhibits unilateral coronal craniosynostosis (**B**, yellow arrowhead, lateral). Note the resultant dysmorphism due to synostosis (**B**, blue asterisk). **D** STRING network summary depiction of known and predicted FUZ protein interactions. Line thickness denotes confidence levels, with thick lines showing highest confidence (0.9). **E** FUZ protein scheme with alignment of relevant Human, Mouse, *Xenopus laevis* and *Drosophila* regions. Predicted longin domains are shown (E, * denotes conserved residues,: denotes residues with strongly similar groups and. denotes residues with weakly similar groups). PHYRE2 secondary structure modelling (E, green spiral = α helix, blue arrow = β sheet).

Here, we report a novel variant in the human *FUZZY* gene as a candidate for craniosynostosis, identified from whole genome sequencing (WGS) of a pair of monozygotic twins with craniosynostosis (case 1 and 2, Table 1, Fig. 1A) and their consanguineous clinically unaffected parents. Just over a handful of pathological variants of *FUZ* have previously been identified (cases 3–6, Table 1); three cases with severe neural tube defects (cases 3–5, Table 1) [8] and one case with the embryonically lethal, short-rib polydactyly syndrome II-like phenotype (case 6, Table 1) [9]. In addition, Zhang et al. 2018 reports the identification of FUZ p.(Arg284Leu), described as an “unsolved case” diagnosed as asphyxiating thoracic dystrophy (ATD) with polydactyly (case 7, Table 1). The fact that so few variants are present in the literature suggests that full loss-of-function changes are lethal, and case 6 further supports this as this early truncation is likely loss-of-function.

**Table 1:** Human variants of *FUZ* and associated phenotypes.

Case	Variant		Type	Zygosity	Reported phenotypes	Source
1 (twinned with 2)	c.851 G > C	p.(Arg284Pro)	Missense	Homozygous	Metopic suture synostosis, Unilateral coronal synostosis, Agenesis of the callosal body	Novel variant
2 (twinned with 1)	c.851 G > C	p.(Arg284Pro)	Missense	Homozygous	Metopic suture synostosis	Novel variant
3	c.155 C > T	p.(Pro39Ser)	Missense	Heterozygous	Premature birth, Myelomeningocele (L5-S2), Chiari II malformation, Hydrocephalus	[8]
4	c.1060 G > T	p.(Asp354Tyr)	Missense	Heterozygous	Lumbosacral myelomeningocele, Chiari II malformation, Hydrocephalus	[8]
5	c.1211 G > A	p.(Arg404Gln)	Missense	Heterozygous	Hemi-myelomeningocele, diastomatomyelia, triventricular hydrocephalus, Chiari II malformation, Moderate transmissive deafness	[8]
6	c.98_111 +9del		In frame frameshift and splice donor removal	Homozygous	Embryonic lethal, post axial polydactyly, short ribs, shortened limbs, heart and kidney defects, midline facial cleft	[9]
7	c.851 G > T	p.(Arg284Leu)	Missense	Unknown	“unsolved case” presenting with asphyxiating thoracic dystrophy and polydactyly	[9]

The human FUZ protein is comprised of 418 amino acids and shares 86.364% sequence homology with mouse FUZ. A number of key protein-protein interactions have been identified, although no specific binding sites have been established (Fig. 1D). FUZ has 3 putative longin domains (Fig. 1E), which function in membrane and vesicular trafficking [10–15]. The *FUZ* gene was first identified as a planar cell polarity (PCP) effector in *Drosophila* [16, 17]. More recent research from our lab and others has highlighted the role of *Fuz* in intraflagellar transport within the primary cilium, a cellular organelle involved in cell-cell signalling, mechano-sensation and signal transduction. Animal models have implicated *Fuz* in Hedgehog (Hh), fibroblast growth factor (FGF) and Wnt signalling pathways;[12, 13, 18, 19] these pathways have all been associated with congenital craniofacial anomalies.

Primary cilia develop in the process of ciliogenesis, where centrioles form basal bodies and dock with the cortex. The microtubules in the cilium are then nucleated from the basal body. Because there is no protein synthesis in the cilium, proteins necessary for cilium function must be actively trafficked to the cilium. Within the cilium this is achieved via the intraflagellar transport (IFT) mechanism [20]. Functional cilia and IFT have both been shown to be crucial for Hh signal transduction [21].

In the absence of Hh ligand, the transmembrane receptor Smoothened (SMO) is inhibited by a second receptor, Patched (PTCH). In this context, downstream GLI transcription factors are processed to repressor forms. Upon ligand binding to PTCH, SMO is activated, allowing relocalisation of the GLI proteins to the cilium. This translocation via the IFT machinery is necessary for processing of GLI2 into a transcriptional activator. A number of craniofacial anomalies have been associated with cilia, IFT and Hedgehog signalling, including holoprosencephaly, cranioectodermal dysplasia, and ATD, mentioned above [22, 23]. In mouse models, mutation in cilia genes, IFT and Hedgehog pathway genes all have severe craniofacial phenotypes, frequently precluding study of the calvaria. Therefore, perturbations of primary cilium associated proteins have not been directly correlated with craniosynostosis [22–24]. However, *Indian Hedgehog* mutants show reduced ossification in the skull [25] and a role in endochondral ossification [26, 27].

In the context of vesicular trafficking, FUZ physically interacts with the small GTPase RSG1 [12, 13], and indeed a mouse knockout of *Rsg1* mostly phenocopies *Fuz* knockouts [28]. During ciliogenesis, *FUZ* is known to interact with Inturned and *WDPCP* (in the ciliogenesis and planar polarity effector (CPLANE) complex) and the IFT-A subunits, which are involved in retrograde intraflagellar transport in the primary cilium [13, 19]. Specifically, IFT-A particles were not localised to the basal body in *fuz* morphant frog embryos. Finally, the CPLANE complex also interacts with chaperonin/CCT complex [13]. This is similar to the BBS6, 10 and 12 complex that helps form the BBSome, another crucial protein complex for primary cilia function. These chaperonin complexes do not enter the cilium but are involved in complex assembly necessary for primary cilium function. Overall, it is clear that FUZ is crucial for the transport of components to the primary cilium and potentially promotes protein complex assembly necessary for downstream cilium formation and function.

Recent work has shown that *Fuz* depletion leads to a gradual loss of the cilium, due to a failure of the retrograde intraflagellar transport machinery [19]. This explains why *Fuz* loss-of-function mouse mutants do not demonstrate early embryonic lethality seen in core ciliogenesis or IFT pathway genes. Nevertheless, we have shown that *Fuz is* required in the cranial neural crest, where *Fuz* mutation leads to a failure to process Gli3 repressors, which was phenocopied in the *Gli3* mouse mutants [29]. This resulted in de-repression of fibroblast growth factor-8 (FGF-8) expression in the head and ectopic expansion of the neural crest domain [29]. This led us to the conclusion that craniosynostosis seen in our mouse models was mimicking that seen in syndromic craniosynostosis models, namely Crouzon and Apert Syndromes, which result from genetic increases in *FGF* signalling.

Our previous studies demonstrated that complete knockout of *Fuz* in a mouse model leads to prenatal lethality, coronal craniosynostosis, micrognathia, facial malformations, eye, and heart defects [12, 24, 29–32]. In this mouse model, we identified several key roles for *Fuz* in skull development: first, mutation of *Fuz* results in an expansion of cranial neural crest, leading to an increase in skeletogenic precursors in the head [29]. Second, using an *Osx-1::GFP-cre* reporter line to lineage label the osteoblast precursors, we showed that the frontal bone mesenchyme was expanded at the expense of the parietal bone [30], which could manifest as craniosynostosis, or an absence of the coronal suture in late gestation animals. These studies identified a clear role for FUZ in early establishment of cell fate and cranial bone tissue boundaries. While mouse *Fuz* is expressed throughout craniofacial structures [31], *FUZ* itself had not previously been directly implicated in osteoblast-specific development or bone formation.

In this study, we report the first craniosynostosis associated variant of *FUZ* and propose a novel function of *FUZ* during the later stages of cranial bone development, using a mouse model to demonstrate that loss of *FUZ* leads to excessive ossification. Furthermore, we find that the novel FUZ p.(Arg284Pro) variant can partially rescue ciliogenesis function in *Fuz* mutant mouse embryonic fibroblasts, suggesting that this variant may reveal cilia dependent and independent functions of FUZ.

## Results

### Novel missense mutation in *FUZ* identified in twins presenting with craniosynostosis

We performed whole genome sequencing and identified a novel homozygous variant in *FUZ* (*c.851* G > C, p.(Arg284Pro)) (Table 1, Fig. 1) in monozygotic twinned, female infants presenting with craniosynostosis. The consanguineous parents (paternal grandfather and maternal grandmother are siblings) are both heterozygous carriers, suggesting that this variant is recessive. Other homozygous variants found that are not present in the control population are presented in Table 2 (12 variants total). SIFT and PolyPhen scores suggested that these other variants were not pathogenic (Supplemental Table 1). The only variant predicted to be involved in craniofacial osteogenesis was *FUZ*. In addition, this novel mutation has not been reported in allele frequency reference databases such as (GnomAD) but is in the same position as an “unsolved case” (p.(Arg284Leu), case 7, Table 1). Moreover, the calculated SIFT and PolyPhen scores were indicative for a pathogenic variant (Supplemental Table 1).

**Table 2:** Homozygous variants found through WGS not present in the wellderly cohort.

Chrom	Position (hg19)	Ref base	Alt base	Gene	Transcript	DNA change	Protein change
chr1	43770666	G	A	*TIE1*	ENST00000372476.8	c.203 G > A	p.(Arg68His)
chr9	90503521	A	C	*SPATA31E1*	ENST00000325643.6	c.4119 A > C	p.(Arg1373Ser)
chr9	95142080	G	A	*CENPP*	ENST00000375587.8	c.503 G > A	p.(Arg168Gln)
chr9	96439169	G	C	*PHF2*	ENST00000359246.9	c.3126 G > C	p.(Gln1042His)
chr10	64913893	A	G	*NRBF2*	ENST00000277746.11	c.779 A > G	p.(Asn260Ser)
chr10	69991419	G	T	*ATOH7*	ENST00000373673.5	c.16 C > A	p.(Pro6Thr)
chr13	32783160	G	T	*FRY*	ENST00000542859.6	c.4189 G > T	p.(Ala1397Ser)
chr13	42623060	C	A	*DGKH*	ENST00000337343.8	c.151 C > A	p.(Leu51Met)
chr14	105415433	T	C	*AHNAK2*	ENST00000333244.6	c.6355 A > G	p.(Met2119Val)
chr19	50312016	C	G	*FUZ*	ENST00000313777.9	c.851 G > C	p.(Arg284Pro)
chr19	55236006	C	T	*KIR3DL3*	ENST00000291860.1	c.5 C > T	p.(Ser2Leu)

Both patients presented at 6 months old with metopic suture craniosynostosis (Fig. 1B–C, yellow arrowhead). Additionally, case 1 (Table 1) presented with unilateral right coronal synostosis (Fig. 1B, yellow arrowhead). While the metopic suture usually fuses between 3–9 months, the coronal suture should remain patent until 20+ years [1]. Both patients also had dilatation of the lateral brain ventricles, with agenesis of the corpus callosum present in case 1 (data not shown). The twins were not reported to have any other phenotypes associated with *FUZ* knockout models, suggesting that this allele may not be a complete loss of function. Further relevant clinical observations are presented in Table 3.

**Table 3:** Clinical observations and phenotypes.

	Case 1	Case 2
Craniosynostosis	Metopic and coronal suture synostosis	Metopic suture synostosis
Eye	Fundoscopy: papilledema OS > OD, no Drusen, no ophthalmologic cause	Fundoscopy: papilledema both eyes
	Skiascopy right eye: S + 3.25 = C-1.0 AS 90;	Skiaskopy right eye: S + 2.50 = C −0.50 AS 6;
	Left eye: S + 6.50 = C-1.0 AS 180	Left eye: S + 2.50 = C-0.50 AS 154
	Ishihara OD 0.5/8 ft OS 0.5/8 ft	Wears glasses
Ear	Prominent ears	Prominent ears
	Right ear larger than left	Frequent ear infections; tubes placed 3 times
Endocrine	N/A	Premature puberty, started hormonal therapy
Skull morphometrics notes	Skull circumference: initially −1, moving to −1.5 SD since age 5 years	Skull circumference: −1SD, moving to −1.5 SD since age 7 years
	Skull length: −3SD	Skull length: −2.5 SD
Other observations	Developmental delay	Developmental delay
	Low Hairline	Low hairline
	Narrow nose	Hypopigmentation caudally of the left nipple
	Ultrasound abdomen aged 6 years: normal	Ultrasound of abdomen aged 6 years: normal
	CT and MRI aged 6 months and MRI aged 18 months: agenesis of the callosal body, stable wide lateral and third ventricles	CT scan aged 6 months: stable dilatation of lateral ventricles
	MRI scan aged 8½ years: unchanged dilatation of ventricles	MRI scan aged 8½ years: dilatation of ventricles, mild agenesis of the callosal body
	Increased intracranial pressure requiring occipital expansion aged 8 years	
Birth observations	At birth 1870 gram, skull circumference 31.5 cm, gestation 36 weeks and 4 days	At birth 2125 grams, skull circumference 31 cm, gestation 36 weeks and 4 days

Key protein-protein interactions of FUZ via STRING analysis indicate roles in PCP signalling (Vangl1, Vangl2, Dvl2 and Intu), Hedgehog signalling (Gli1, Gli2, Gli3 and Sufu) and in the CPLANE complex (Intu, Rsg1 and Wdpcp) (Fig. 1D). Within *FUZ*, the arginine residue at amino acid position 284 is conserved across human, *Xenopus*, mouse, and *Drosophila* and lies adjacent to the C-terminal longin domain (Fig. 1E), which is thought to mediate vesicular trafficking [10–12]. This mutation is predicted to be deleterious (CADD score 25.4 (CADD Exome (1.6.1)). Arg284 lies outside of the predicted α helices or β sheets in FUZ (Fig. 1, E) suggesting that any structural change from the Arg284Pro variant is likely to be in the tertiary structure of the protein rather than affecting secondary structure. Other reported *FUZ* gene variants (Table 1) are either missense or truncating and will be discussed further in discussion. However, none are associated with craniosynostosis.

### The role of FUZ during craniofacial ossification

**Fig. 2:**
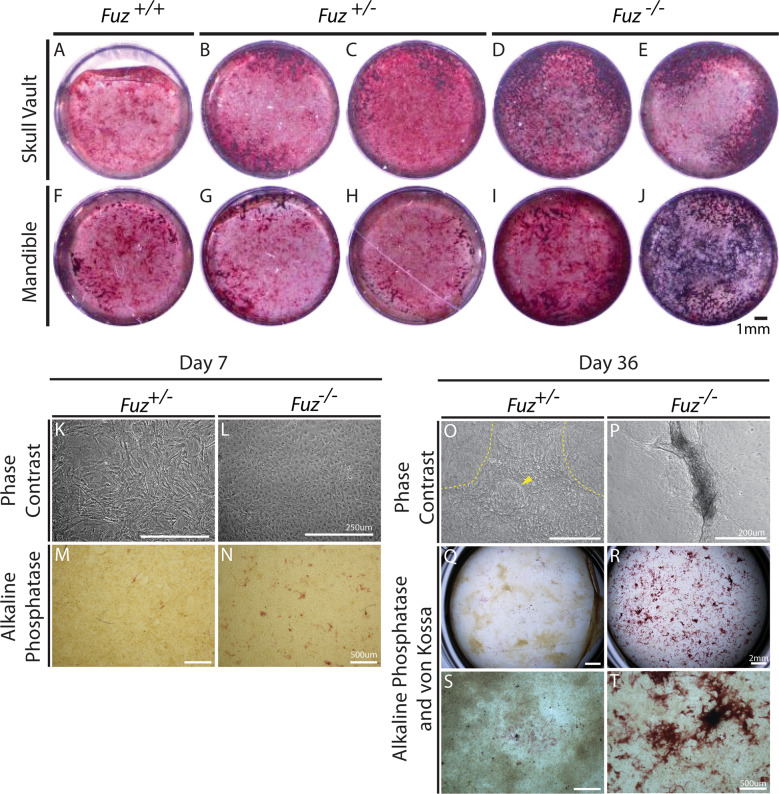
*Fuz* mutant cells exhibit increased mineralisation in vitro. **A**–**J** Skull vault and mandibular bones were dissected from E18.5 foetuses. Isolated osteoblasts were cultured for 14 days and assayed for alkaline phosphatase activity (red) and mineral deposition (black, Von Kossa staining). Increased mineralisation was seen in *Fuz*^*−/−*^ cultures (skull vault; **D**, **E**, mandible; **I**, **J**) compared to controls (*Fuz*^*+/+*^ (Skull vault; **A**, Mandible; **F**) and *Fuz*^*+/−*^ (Skull vault; **B**, **C**, Mandible; **G**, **H**). **K**–**T** Primary mouse embryonic fibroblasts were isolated from E12.5 animals and cultured in osteogenic media for seven days (**K**–**N**) or 36 days (**O**–**P**). At seven days, controls were fibroblastic (**K**) while mutant cells showed a cobblestoned appearance (**L**) with increased alkaline phosphatase expression (**N**). By day 36, cellular condensations were seen in controls (**O**, yellow arrowhead and dashed lines) whereas mutants exhibited refractive, mineralised nodules (**P**). No nodules were seen in control cultures. Mutant cultures had vastly increased alkaline phosphatase expression compared with controls (compare **R** & **T** to **Q** & **S**). Mutants showed some alkaline phosphatase positive nodules (red) co-staining with Von Kossa stain (black) (compare **T** to **S**). Scale bars as indicated.

To date most research has focused on the requirements of *FUZ* during neural crest induction, and toward implications for patterning of the craniofacial skeleton. Specific roles during osteogenesis or bone formation have not been explored. Given the craniosynostosis observed in these patients, we set out to test whether mouse *Fuz* is required during craniofacial osteoblast mineralisation. Homozygous *Fuz* mutant mice exhibit 100% prenatal lethality with a progressive loss from embryonic day 13.5 (E13.5) onwards. Therefore heterozygous *Fuz*^*+/−*^ mice were intercrossed to generate control wild-type, heterozygous and homozygous null animals, which were collected at embryonic day 18 (E18.5), just prior to birth. Primary osteoblasts were isolated from the skull vaults and mandibles for culturing in a mineralisation assay (Fig. 2). Pre-osteoblasts were assessed based on alkaline phosphatase levels (red staining), while von Kossa staining was used to determine overall mineralisation levels (black staining). Interestingly, *Fuz* mutant osteoblasts from both skull vault and mandible showed increased mineralisation compared to the wildtype and heterozygous controls (Fig. 2, black staining in D, E, I and J compared to A-C and F-H). This suggests that the mineralisation potential of *Fuz* mutant osteoblasts is increased.

Because the severe prenatal lethality seen in the *Fuz*^*−/−*^ mice limited our access to calvarial osteoblasts, we then turned to mouse embryonic fibroblasts (MEFs) to further confirm the pro-osteogenic phenotypes. MEFs can be induced to undergo osteogenesis when challenged with media containing bone morphogenetic protein-2 (BMP-2). Within 7 days, *Fuz* mutant cells had a more cobblestoned appearance compared to the more fibroblastic wildtype cells (Fig. 2K, L). In addition, an increase in the area of alkaline phosphatase positive cells was seen (red staining Fig. 2M and N). By 36 days in culture, the appearance of the cultures was very different, with *Fuz* mutant cells showing many refractive, mineralised alkaline phosphatase positive nodules (Fig. 2O–T).

### FUZ p.(Arg284Pro) can partially rescue FUZ mutant ciliogenesis

**Fig. 3:**
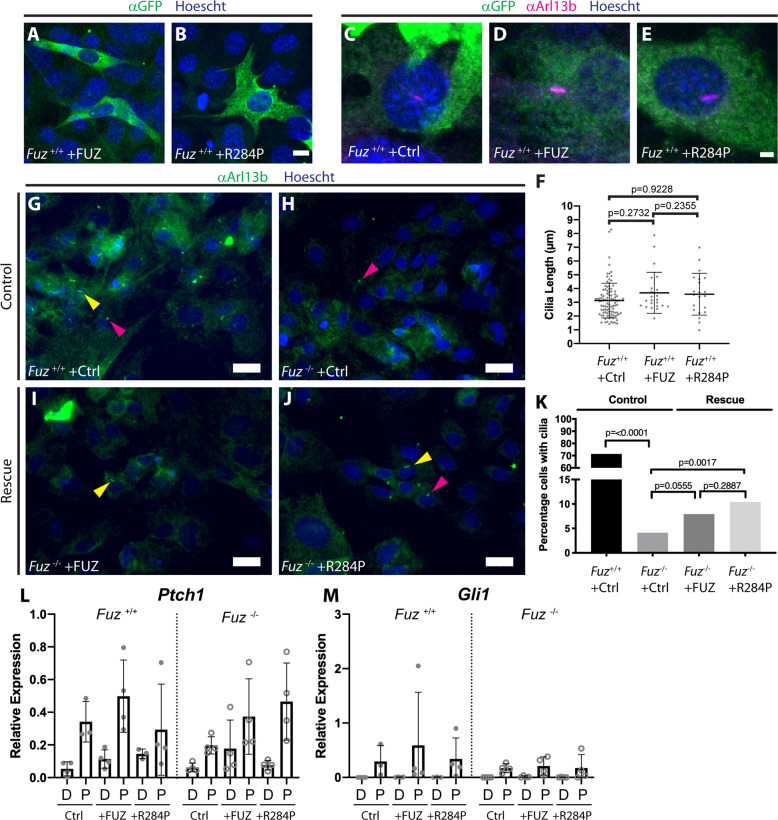
FUZ p.(Arg284Pro) partially rescues in vitro ciliogenesis phenotype. Both GFP-FUZ-FLAG (+FUZ) or GFP-FUZ p.Arg284Pro-FLAG (+R284P) transfected in immortalised wildtype MEFs show similar cytoplasmic localisation (**A**–**B**, anti-GFP (green)). Transfection of GFP-FUZ-FLAG (+FUZ) or GFP-FUZ p.Arg284Pro-FLAG (+R284P) resulted in no significant change in primary cilium length (measured from anti-anti-Arl13b staining (magenta), quantified in (**F**). Rescue experiments were performed in primary MEFs. Ciliary axoneme staining (**G**–**J**, anti-Arl13b, green) revealed significantly decreased numbers of cilia present in mutant (**H** and **K**, 3% of cilia positive cells) compared to control GFP transfected cells (**G** and **K**, 70% cilia positive cells, *p* = <0.0001, (**K**). Mutant cells transfected with FUZ (**I**) or FUZ p.(Arg284Pro) constructs (**J**) exhibited 4% (**K**, *p* = 0.0555) and 7% (**K**, *p* = 0.0017) increase in cilia number compared to mutant cells transfected with GFP. Cilia were present as dots (**G**–**J**, pink arrowhead) or lines (**G**–**J**, yellow arrowhead) and were both included in quantification. RT-qPCR relative expression for Hedgehog transcriptional readouts *Ptch1* and *Gli1* (**L** and **M**) from immortalised MEFs. Data grouped by genotype as indicated with DMSO [D] or 2 µM Purmorphamine [P] treatments and no transfection (Ctrl), transfection of GFP-FUZ-FLAG (+FUZ) or GFP-FUZ p.Arg284Pro-FLAG (+R284P). Statistics and *P* values (**F**) are student’s *T* tests and (K) are Fisher’s exact tests comparing conditions as indicated. Scale bars (**A**, **B**) = 10 µm, (**C**–**E**) = 3 µm, (**G**–**J**) = 50 µm.

If the function of FUZ is impaired by the p.(Arg284Pro) variant, then we might expect to see that protein localisation, ciliogenesis, or signalling function of the primary cilium is also impaired. We expressed GFP-fused versions of both variants in wildtype MEFS in order to test their localisation, and found that both forms localised to the cytoplasm (Fig. 3A, B). We then examined their effects on ciliogenesis. Normally, MEFs cultured in reduced serum media will each generate a single primary cilium. Using this assay we checked whether overexpression of *FUZ* could increase the length of the cilium as previously reported in MDCK cells;[8] however, we did not observe any increase in length, based on staining for the ciliary marker Arl13b (Fig. 3C–F).

We then used MEFs from *Fuz* knockout mice to assess whether p.(Arg284Pro) variant could rescue ciliogenesis, due to the observation that MEFs from *Fuz*^*−/−*^ mutant animals very rarely generate cilia (3% (Fig. 3H and K, *n* = 315, compared to 70% wildtype MEFs Fig. 3G, K, *n* = 168). *Fuz*^*−/−*^ MEFs were transfected with either a control GFP plasmid, a full-length wild-type *FUZ* construct, or the variant *FUZ-p.(Arg284Pro)* construct and assessed for cilia formation. Transfection efficiencies were low; however, the wild-type FUZ construct rescued ciliogenesis in a significant proportion of cells (7% seen in Fig. 3I and K, *n* = 277, *p* = 0.0555 compared to *Fuz*^−/−^). Similarly, transfection with the FUZ-p.(Arg284Pro) variant construct increased the proportion of cells with a cilium to 10% (Fig. 3J, K, *n* = 403, *p* = 0.0017 compared to *Fuz*^−/−^). Altogether, this suggested that the p.(Arg284Pro) variant could function similarly to the wild-type protein during ciliogenesis.

### Inefficient transduction of the Hedgehog signal response by FUZ p.(Arg284Pro)

While FUZ p.(Arg284Pro) appeared to restore some ciliogenesis, this is relatively unsurprising since the *Fuz*^*−/−*^ mice do initially have functional cilia. A defect in Hedgehog signal transduction arises subsequently, likely due to poorly functioning of retrograde IFT leading to cilia degeneration [19]. To address the ciliary function of FUZ p.(Arg284Pro) we compared the ability of wildtype human FUZ and the FUZ p.(Arg284Pro) variant to increase Hh responsiveness in both control and mutant *Fuz* MEFs. We performed RT-qPCR for two transcriptional targets of Hedgehog, *Patched-1 (Ptc1)* and *Gli1. Ptc1* is thought to be an immediate early response to Hh activation, while *Gli1* requires a sustained and higher Hh cue. First, we note that the wildtype MEFs respond robustly to treatment with 2 µM purmorphamine, which binds to Smoothened and mimics Hh ligand activation (Figure L and M, *Fuz*^*+/+*^, P, Ctrl). In contrast, *Fuz*^*−/−*^ MEFs do respond, but less robustly (Fig. 3L, M). In control MEFs, transfection with wildtype *FUZ* increases expression of *Ptch1* over baseline levels. Transfection of the *FUZ* p.(Arg284Pro) variant also increases target gene activation, but less than the wild-type (Fig. 3L–M, *Fuz*^*+/+*^, P, Arg284Pro). The variability in these experiments is likely due to transfection efficiencies; therefore, future experiments should be performed using stable transgenic lines, with further confirmation by immunolocalisation of Hh pathway effectors. Nevertheless, our observations support the idea that normal MEFs carrying the FUZ p.(Arg284Pro) variant are less efficient when transducing Hedgehog signals. In *Fuz*^*−/−*^ MEFS, we found that transfection of either human *FUZ* variant was sufficient to increase both *Ptc1* and *Gli1* transcription; however, response to FUZ p.(Arg284Pro) was increased with regards to *Ptc1* while we saw almost no response in *Gli1*.

## Discussion

The genetic aetiology of craniosynostosis is poorly understood. Here, we report a novel craniosynostosis associated mutation in *FUZ* (*c.851* G > C, p.(Arg284Pro)). We found that loss of *Fuz* resulted in increased mineralisation in both in vitro embryonic primary osteoblast cultures and in fibroblasts undergoing an osteogenic challenge. A direct effect of *FUZ* mutation in the late stages of bone development and mineralisation has not been reported before. In addition, the novel variant also partially rescued the loss of primary cilia phenotype observed in mutant MEFs [8, 12]. From this we can make the following conclusions: that FUZ is a negative regulator of osteoblast mineralisation and that the p.(Arg284Pro) variant is not a complete loss of function during cilia formation. However, this variant may be inefficient when transducing Hh signalling.

No previous reports have implicated changes in human *FUZ* in craniosynostosis. However, variations in *FUZ* have been found in patients with neural tube defects. Three pathogenic variants were previously investigated (Table 1, case 3-5). The p.(Pro39Ser) variant (case 3, Table 1) exhibited increased cell migration in scratch/wound assays but no effect on ciliogenesis. The opposite was true for the p.(Arg404Gln) variant (case 5, Table 1) where ciliogenesis was impacted but cell migration was not. Finally, the p.(Asp354Tyr) variant exhibited both increased cell migration and defective ciliogenesis (case 4, Table 1). This puts forward the possibility that the C-terminal domain of FUZ (including residues 354 and 404) is necessary during ciliogenesis. We suggest that this functional region excludes the Arg284 residue due to the partial rescue of ciliogenesis seen in our experiments (Fig. 3). In the longer term, it will be of interest to investigate ossification ability of the p.(Pro39Ser) variant in comparison to our p.(Arg284Pro) data.

More recently, a mutation leading to an in-frame frameshift and splice donor site removal was reported in *FUZ*, leading to an early truncation and likely loss of function [9]. This mutation led to prenatal lethality and presented with a phenotype closely resembling short rib polydactyly syndrome type II (OMIM #613091). The phenotypes observed were small chest and short limbs with polydactyly, cardiac and kidney defects, and a midline facial cleft, most similar to ciliogenesis variants. As this change likely results in a complete loss of function, (due to the early stop), we would expect both ciliogenesis and ossification phenotypes. In support of this, the phenotypes seen in this patient more closely resembles those found in mouse mutants with a complete loss-of-function [12, 24, 31]. Zhang et al. 2018 [9] also reports a ‘unsolved case’ variant of *FUZ* at p.(Arg284Leu). If this mutation is the true cause of this ATD case then this supports strongly the hypothesis that variants at this locus can cause ciliopathic phenotypes.

This work is the first demonstration that FUZ can act as a negative regulator of osteoblast mineralisation. We propose that ciliopathic function may affect the craniofacial skeleton due to a requirement for cilia dependent signalling at several stages during osteogenesis. While a requirement for cilia are well-established during induction of the neural crest precursors of the cranial skeleton, the later requirements during bone formation and mineralisation are less appreciated. The reported gene variants of FUZ including the novel p.(Arg284Pro) variant provide us with an opportunity to dissect the sequential uses of FUZ and CPLANE proteins during development of the craniofacial skeleton. In the case of FUZ, these variants could be introduced to primary cells or cell lines using CRISPR, or virally into mutant cells, and subsequently used to test specific functions, which in the long term could separate the functional domains controlling vesicle trafficking, osteoblast mineralisation and ciliogenesis functions of FUZ.

Based on animal models and human case studies, it is clear that genetic mutations affecting the structure and function of the primary cilium can result in developmental abnormalities and skeletal dysplasias [33]. These often affect the limbs with polydactyly and the face with cleft lip/palate, micrognathia, facial width abnormalities and craniosynostosis. Several ciliopathies that predominantly affect the craniofacial skeleton present with craniosynostosis [22]. Together, this suggests an overlap between ciliopathic syndromes and craniosynostosis [29, 30] and attributes a subset of these to Hedgehog signalling changes.

Hh signalling can affect both osteoblast differentiation and mineralisation. Indian Hedgehog (IHH) signalling is necessary for the early differentiation of osteoblasts, with upregulation of Hh signalling via the small molecule purmorphamine resulting in increased commitment to osteoblastic maturation [34]. A loss at this stage could leave osteoprogenitors in a proliferative and undifferentiated state, effectively increasing the number of precursors able to differentiate and mineralise. In contrast, loss of Hh signalling, via a conditional knockout of the *Smoothened* receptor in mature osteoblasts, results in increased bone mass and a disruption in bone homeostasis [35]. Thus, it is evident that Hh signalling is necessary at several key time points during skeletal development and that loss of *FUZ* may reduce the Hh signalling capacity via the primary cilium at these critical stages.

However, simple non-syndromic craniosynostoses are often surgically treated and whether there is a genetic aetiology is not assessed. These cases could be caused by partial loss of function variants in multifunction genes, like *FUZ*. Where complete loss of function would cause systemic anomalies; here, a missense or partial loss of function mutation may affect a specific functional region of the protein giving a ‘microform’ presentation of a syndrome. Linking genetic variants to the functional regions of proteins will help us understand the phenotypic manifestation of the syndrome, as well as the relative severity. This will help with genetic counseling, diagnosis and may determine what future follow-up is required.

## Materials and methods

### Sequencing

Whole genome sequencing (WGS) was performed on DNA from blood by Complete Genomics, (Mountain View, CA, USA) [36]. Variants were annotated using NCBI build GRCh37/hg19 and dbSNP build 137. Data were analyzed using cga tools version 1.8.0. An autosomal recessive disease model was tested. The analysis was restricted to novel non-synonymous variants, variants disrupting a splice site (±two basepairs), and insertions or deletions in the coding sequence (±50 bp). The remaining variants were analyzed with Annovar [37] and OpenCravat [38] to get an indication of the pathogenicity and allele frequency, and compared to those present in the wellderly cohort [39]. The variant identified was described according to HGVS nomenclature [40], using reference sequence NM_025129.5, on GRCh37/hg19 and was submitted to the Leiden Open Variation Database. The variant identified by WGS was validated by dideoxy-sequence analysis.

### Mouse lines and animal husbandry

*Fuz* mutants (MGI:3531090) in this study were previously reported [12]. All animal work was carried out in accordance with UK Home Office regulations under the project licence P8D5E2773 held by KJL. Immortalised MEFs were derived from another previously reported *Fuz* mutant line [8].

### Cell culture

Primary mouse embryonic fibroblasts (MEFs) were isolated from E12.5 embryos using standard procedures (Fig. 2, Fig. 3 G-K), while immortalised MEFs have been previously described (used in Fig. 3 A-E and L, M) [8]. MEFs were passaged every 3-4 days and primary MEFs were used up to passage 5. DNA transfections were performed with Lipofectamine LTX or 2000 reagent (Thermofisher) per manufacturer’s protocol. For cilia induction FBS content of MEF media was reduced to 0.5% for 48 hours.

Primary osteoblasts were isolated from the dissected calvaria and mandibles of staged E18.5 mouse embryos. Cells were plated in a 24-well plate in primary osteoblast growth media (α-MEM (Lonza), 10% batch tested osteogenic FBS, 1X ABAM, 1X L-glutamine) and incubated at 37 °C with 5% CO_2_. When confluent, cells were trypsinised and chips allowed to sediment. The resulting cell solution was then passed through a 40um cell strainer. Cells were resuspended to 40,000cells/well, plated into a 48 well plate and cultured for 10 days in osteoblastic mineralisation medium (primary osteoblast growth media, 50ug/ml ascorbic acid, 5 mM β-glycerophosphate). For MEF to osteoblast differentiation primary MEFs were cultured in osteoblastic mineralisation media +50 ng/ml BMP2. Standard protocols for alkaline phosphatase staining and subsequently Von Kossa staining were then carried out.

### Reverse transcription-quantitative PCR

RNA was extracted using Trizol (Sigma), followed by cDNA synthesis according to conventional methods [41]. The following quantitative PCR primers were used: mouse β-actin: for-CTAAGGCCAACCGTGAAAAG3ʹ, rev-ACCAGAGGCATACAGGGACA; mouse *Patched-1 (Ptch1)* for-AAGCCGACTACATGCCAGAG, rev-AAGGGAACTGAGCGTACTCG; mouse *Gli1* for- CAGGGAAGAGAGCAGACTGAC, rev CGCTGCTGCAAGAGGACT.

### Immunofluorescent antibody staining

Primary MEFs were fixed in 100% Methanol for 15 mins on ice. After washing (1% BSA, 0.1% Tween20 in 1X PBS) cells were permeabilised with 0.5% TritonX-100 (in 1X PBS) and blocked in (3% BSA, 10% Goat serum, 0.1% Tween20, in 1X PBS) for 1 hour at room temperature. Anti-ARL13b primary antibody (1:500, Proteintech, 17711-1-AP) was incubated in blocking buffer overnight at 4 °C.

Immortalised MEFs were fixed in 4% PFA for 10 mins at room temperature and stained with anti-GFP (1:500, Abcam, ab13970) and anti-Arl13b (as before) for 1 hour at room temperature. Coverslips were then washed and incubated in anti-rabbit 488, anti-Chicken 488 or anti-Rabbit 568 secondary (1:500, Invitrogen, A11008, A11039 and A11011) for 2 hours at room temperature. Nuclei were stained with Hoechst 33342 (Sigma) 1:1000 (20 mg/ml stock). Plates were imaged using standard inverted epifluorescent microscope (Zeiss) or confocal (Nikon A1R). Image processing was carried out in FIJI (ImageJ) [42]. Cilia length was measured using maximum intensity projections from Arl13b staining in GFP positive cells (indicating transfection).

### Protein predictions and secondary structure prediction

STRING queries (https://string-db.org/) were rooted on human FUZ protein identifier ENSP00000313309 showing functional and physical protein associations. Human FUZ amino acid sequence was analysed using PHYRE2 (an ab initio and homology modelling tool) [43]

## Supplementary information

Supplemental Table 1

## Data Availability

Data available within the article or upon reasonable request. The p.(Arg284Pro) variant is reported in the Leiden Open Variation Database (https://databases.lovd.nl/shared/individuals/00375537).
